# Effects of Delayed-Release Olive Oil and Hydrolyzed Pine Nut Oil on Glucose Tolerance, Incretin Secretion and Appetite in Humans

**DOI:** 10.3390/nu13103407

**Published:** 2021-09-27

**Authors:** Karina V. Sørensen, Mads H. Kaspersen, Jeppe H. Ekberg, Annette Bauer-Brandl, Trond Ulven, Kurt Højlund

**Affiliations:** 1 Steno Diabetes Center Odense, Odense University Hospital, 5000 Odense, Denmark; karina.vejrum.sorensen@rsyd.dk; 2 Department of Clinical Research, University of Southern Denmark, 5000 Odense, Denmark; 3 Department of Physics, Chemistry and Pharmacy, University of Southern Denmark, 5230 Odense, Denmark; mmk@sdu.dk (M.H.K.); annette.bauer@sdu.dk (A.B.-B.); tu@sund.ku.dk (T.U.); 4 Department of Drug Design and Pharmacology, University of Copenhagen, 2100 Copenhagen, Denmark; jpe@sund.ku.dk; 5 Center for Basic Metabolic Research, University of Copenhagen, 2200 Copenhagen, Denmark

**Keywords:** G-protein-coupled receptors, pine nut oil, pinolenic acid, olive oil, 2-oleoylglycerol, incretins, glucose tolerance, appetite

## Abstract

Background: To investigate the potential synergistic effects of olive oil releasing 2-oleoylglycerol and hydrolyzed pine nut oil containing 20% pinolenic acid on GLP-1 secretion, glucose tolerance, insulin secretion and appetite in healthy individuals, when delivered to the small intestine as potential agonists of GPR119, FFA1 and FFA4. Methods: Nine overweight/obese individuals completed three 6-h oral glucose tolerance tests (OGTTs) in a crossover design. At −30 min, participants consumed either: no oil, 6 g of hydrolyzed pine nut oil (PNO-FFA), or a combination of 3 g hydrolyzed pine nut oil and 3 g olive oil (PNO-OO) in delayed-release capsules. Repeated measures of glucose, insulin, C-peptide, GLP-1, GIP, ghrelin, subjective appetite and gastrointestinal tolerability were done. Results: PNO-FFA augmented GLP-1 secretion from 0–360 min compared to no oil and PNO-OO (*p* < 0.01). GIP secretion was increased from 240–360 min after both PNO-FFA and PNO-OO versus no oil (*p* < 0.01). Both oil treatments suppressed subjective appetite by reducing hunger and prospective food consumption and increasing satiety (*p* < 0.05). Conclusions: In support of previous findings, 6 g of delayed-release hydrolyzed pine nut oil enhanced postprandial GLP-1 secretion and reduced appetite. However, no synergistic effect of combining hydrolyzed pine nut oil and olive oil on GLP-1 secretion was observed. These results need further evaluation in long-term studies including effects on bodyweight and insulin sensitivity.

## 1. Introduction

The free fatty acid receptor-1 (FFA1), free fatty acid receptor-4 (FFA4) and G-protein-coupled receptor-119 (GPR119) are G-protein-coupled receptors (GPCRs) activated by certain lipid metabolites. These receptors have caught the interest of researchers due to their modulatory effects on important metabolic responses including pancreatic insulin secretion, gastrointestinal secretion of incretin hormones, appetite regulation, insulin sensitization and anti-inflammation, effects with beneficial prospects for metabolic diseases such as obesity and type 2 diabetes.

FFA1 and FFA4 are activated by medium- and long-chain fatty acids [1], whereas GPR119 is known to respond to various lipid ligands, including 2-monoacylglycerols (2MAGs) [2,3]. FFA1, FFA4 and GPR119 are expressed in the gastrointestinal tract in enteroendocrine cells (EC) [2,4,5,6]. Activation of the receptors in ECs has been shown to stimulate the secretion of incretins such as glucagon-like peptide-1 (GLP-1) and glucose-dependent insulinotropic peptide (GIP) [2,4,5,6], although this effect has been questioned for FFA4 [7]. The incretin hormones are capable of enhancing glucose-stimulated insulin secretion (GSIS) [8]. Furthermore, GLP-1 has additional effects including appetite suppression leading to subsequent loss of body weight when used in pharmacological doses [8,9]. FFA1 and GPR119 are also present on pancreatic beta-cells where they enhance GSIS [10,11]. The potential of stimulating FFA1 was convincingly demonstrated in a phase 3 trial, where the pharmaceutical FFA1 agonist TAK-875 was able to reduce glycated hemoglobin levels in patients with type 2 diabetes. However, further studies of this drug were terminated due to liver toxic side effects [12]. FFA4 is also present on ghrelin secreting cells, and it has been demonstrated that FFA4 activation inhibits ghrelin secretion in humans [13]. Ghrelin is an orexigenic hormone playing a central role in appetite control [14]. Thus, a reduction in ghrelin levels can potentially reduce appetite and decrease energy intake and body weight. Moreover, in animal and cell studies, the activation of FFA4 on macrophages and in adipose tissue has been reported to reduce inflammation and improve insulin sensitivity [15,16].

In a recent study, screening of 46 fatty acids demonstrated pinolenic acid to be the most potent dual agonist of human FFA1 and FFA4 in vitro [1]. Moreover, pinolenic acid and Siberian pine nut oil (containing 20% pinolenic acid) were shown to reduce glucose levels compared to maize oil during an oral glucose tolerance test (OGTT) in mice, with a superior effect of pinolenic acid [1]. We further evaluated these effects in a clinical study testing the effects of delayed-release hydrolyzed pine nut oil versus genuine unhydrolyzed pine nut oil prior to an OGTT [17]. While hydrolyzed pine nut oil caused only a small reduction in glucose levels from 0–120 min, we observed a more pronounced increase in circulating GLP-1 levels and a concomitant reduction in ghrelin levels from 120–240 min compared with no oil control treatment. Moreover, subjective appetite was reduced. Thus, hydrolyzed pine nut oil delivered to the small intestine by delayed-release capsules may be a beneficial functional dietary lipid able to stimulate GLP-1 secretion and reduce ghrelin levels late in the postprandial state causing an appetite reducing effect.

GPR119 in transfected cell lines can be activated by a number of 2MAGs including 2-oleoylglycerol (2OG) [3], comprising an oleic acid attached to the sn-2 position of glycerol. Though oleoylethanolamide is a more potent endogenous agonist of GPR119, the much higher abundancy of 2OG (and other 2MAGs) in the intestine, formed as metabolic product during digestion and absorption of triglycerides, outweighs the lower potency of 2OG and makes it a better dietary candidate for activating GPR119 [3]. Purified 2OG (2 g) administered through a nasojejunal tube and also ordinary oral intake of olive oil (19 g, liberating 7.7 g 2OG upon complete digestion) have been shown to effectively stimulate GLP-1 release in humans [18,19,20]. Interestingly, it was recently demonstrated, using specific tool compounds in a cell model, that simultaneous activation of GPR119 and FFA1 act in synergy to enhance enteroendocrine GLP-1 secretion beyond the combined individual responses [7].

The aim of this study was to evaluate the potential synergistic effect of combined activation of FFA1, FFA4 and GPR119 by intake of delayed-release hydrolyzed pine nut oil (containing pinolenic acid) and olive oil (liberating 2OG upon digestion) on incretin hormones, glucose tolerance, insulin secretion, insulin sensitivity and appetite in vivo in healthy humans.

## 2. Materials and Methods

The study was performed at Steno Diabetes Center Odense, Odense University Hospital, Denmark, and was approved by the Regional Committees on Health Research Ethics for Southern Denmark (S-20150060). Moreover, it was conducted in accordance with the Helsinki Declaration.

### 2.1. Participants

Participants were included in the study based on the following criteria: age from 40–70 years, BMI 27.5–40 kg/m^2^, normal glucose tolerance (2-h plasma glucose <7.8 mmol/L), normal screening blood samples (within Danish reference ranges) including markers of kidney and liver function, cholesterols, triglycerides and hematology. Participants had to be healthy with a normal blood pressure (140/90 mmHg), no need for prescriptive medicine, no chronic diseases including gastrointestinal diseases or previous gastrointestinal surgery, no first-degree relatives with diabetes, and no food allergies of importance. Moreover, they had to be non-smokers with a stable body weight (<3 kg change within three months prior) and no intake of dietary supplements (within 1 month prior) or adherence to any type of restrictive diet (calorie restriction, vegan diet, etc.). All participants gave written informed consent prior to any study related procedures.

Out of eleven pre-screened participants invited to the initial screening OGTT, two were excluded due to impaired glucose tolerance. Thus, nine participants completed the study protocol. Baseline characteristics are shown in Table 1.

### 2.2. Study Design

The study was a crossover trial with three different arms. The first arm was a 6-h OGTT (75 g glucose) with no oil intake prior to the test corresponding to the initial screening OGTT for the nine participants. Hereafter, two additional OGTTs were completed in a randomized order (www.randomizer.org, 2 May 2018) consuming either 6 g of hydrolyzed pine nut oil (PNO-FFA) alone or 3 g hydrolyzed pine nut oil combined with 3 g olive oil (PNO-OO), in both cases together with 160 mL of water 30 min prior to the OGTT. The same amount of water was also consumed 30 min prior to the screening/control OGTT. A washout period of one to four weeks between the OGTTs was applied (Supplementary Figure S1). During all OGTTs sampling of blood was done at −30, −15 and 0 min, and every half hour until 240 min, and additionally at 300 and 360 min. Prior to all experimental days, participants consumed a self-made standard evening meal with no calorie restriction and were instructed to fast from 10 PM after intake of the meal (a small amount of water was allowed). Furthermore, participants were instructed to abstain from physical exercise and alcohol consumption 48 h prior to the experimental days and to attend the research facility by car or a non-strenuous way of transportation. All participants were instructed to maintain their habitual lifestyle until the end of the study. The study was performed from June to December 2018.

### 2.3. Subjective Appetite and Gastrointestinal Tolerability

Appetite was assessed by the use of visual analog scale (VAS) questionnaires including hunger, satiety, fullness, prospective food consumption and overall well-being [21]. The degree of each symptom was indicated on a 100 mm horizontal line, where 0 and 100 mm indicated the extreme feelings related to the symptom in question. At every OGTT appetite questionnaires were completed at −30, −15, 0, 15, 45, 60 min and every half hour until 240 min and hereafter at 300 and 360 min.

Similarly, we used VAS questionnaires to measure gastrointestinal tolerability by assessing the following symptoms: nausea/vomiting, bloating, flatulence, constipation, diarrhea and abdominal pain [22]. Questions were formulated to reflect the acute setting of the test. Again, 0 mm and 100 mm indicated the extreme degree of the symptom in question, that is: no degree of the symptom or a severe degree. This was done prior to the OGTT, after the OGTT and at 8:00 p.m. on the same evening and 8:00 a.m. the morning after.

### 2.4. Indices of Insulin Sensitivity and Beta-Cell Function

OGTT based insulin sensitivity was determined by BIGTT_Si_ (0, 30 and 120 min) [23] and MATSUDA (0, 30, 60, 90 and 120 min) [24], and beta-cell function was determined by BIGTT_AIR_ (0, 30 and 120 min) [23] and CIR_30 min_ [25].

### 2.5. Analysis of Blood Biomarkers

Plasma glucose was analyzed by an ABL800 FLEX blood gas analyzer, and serum insulin and C-peptide were analyzed on a Cobas e 411 with an electrochemiluminescence immunoassay (ECLIA). Insulin intra- and inter-assays had a CV% of 1.9–2.0 and 2.5–2.6, respectively. The C-peptide intra- and inter-assay CV% were 1.3–4.6 and 1.8–5.0, respectively. Plasma free fatty acids (FFA) were analyzed using the in vitro enzymatic colorimetric method assay NEFA-HR(2) (Wako Chemicals, GmbH, Neuss, Germany). Plasma GLP-1 was assessed by the Total GLP-1 (ver. 2) Assay Kit from Meso Scale Discovery. Plasma GIP and ghrelin were assessed by using the Human GIP (Total) or Human Ghrelin (Total) ELISA Kit from Millipore. Intra- and inter-assay CV% of the GIP kit was 3.0–8.8 and 1.8–6.1, and the intra- and inter-assay CV% of the ghrelin kit was 0.90–1.91 and 5.18–7.81.

### 2.6. Intervention Oils

The intervention oils used in this study were extra virgin olive oil (Piccardo & Savoré, Chiusavecchia, Italy) and free fatty acids of Siberian pine nut oil (Siberian Pine Nut Oil, Port Charlotte, FL 33952, USA). The later which fatty acid composition was analyzed and can be reviewed in a previous study by our group [1]. Hydrolysis of the pine nut oil was conducted as previously described [17]. Both oils were manually encapsulated in white semitransparent DRcaps™ from Capsugel^®^ (size 00 batch: 5332311). These are resistant to acid and made of hydroxypropyl methylcellulose with delayed release properties. According to the manufacture, release is postponed approximately 45 min when using dry substance filling [26]. In a previous study, we conducted our own in vitro evaluation of the release profile of capsules filled with PNO-FFA and olive oil, and found an immediate but slow release of capsule content over 240 min [17]. Both intervention doses were 6 g of oil in total, which amounted to ten capsules (corresponding to ~225 kJ). Intervention doses were selected based on several factors, (1) the amount should be fit for long term studies and therefore realistic and safe to consume over a longer period of time, and potentially later as a marketable product, (2) hydrolyzed pine nut oil has not previously been consumed in larger doses, therefore due to safety concerns we used small doses and (3) hydrolyzed pine nut oil has been investigated in doses of 2–3 g in earlier studies, and shown to have effects on appetite regulating factors including gut hormone secretion and subjective appetite [27,28]. Subjects were blinded to oil type; however, a slight color difference was apparent for the individual oil types on the PNO-OO treatment. Oil capsules were kept at a temperature of 5 °C and stored <3 months.

### 2.7. Outcomes and Statistics

Treatment outcomes on glucose, insulin, C-peptide, GLP-1, GIP, ghrelin and FFA were evaluated and compared by calculating the total area under the curve (AUC) during the full six hours of testing (AUC_total_) and for two hour segments (AUC_0–120_, AUC_120–240_, and AUC_240–360_). Similarly, VAS treatment responses on appetite were calculated as AUCs for the full period of testing and two-hour segments. All AUCs were calculated by the use of the trapezoidal rule [29]. VAS treatment responses on gastrointestinal tolerability were assessed as changes during the OGTTs and treatment differences at 8 PM on the same evening after the OGTTs and at 8 AM the morning after the OGTTs.

Mixed model linear regression was used to compare AUCs of blood parameters and appetite. Model fit was assessed by qq-plots of the residuals and plots of residual versus fitted values. If these were unfit, data were transformed by the use of natural logarithm. We experienced a few missing data points on GIP (6/834), several on ghrelin (21/834) and a few on appetite (5/2025). For GIP and ghrelin, in cases with only one missing data point from 30 min to 300 min (GIP 2 cases, ghrelin 7 cases), these were omitted in the calculation of the AUC, corresponding to imputing the mean value of results from the adjacent data points. For ghrelin, three subjects missed three or more consecutive results between 30 and 240 min of the test in one out of the three experimental arms. These were excluded prior to analysis, which meant that the PNO-FFA treatment was reduced to seven subjects and PNO-OO to six subjects. Moreover, data points missing at 360 min (GIP 3 cases, ghrelin 2 cases) were missing during analysis and model estimations were used in these cases. Changes in gastrointestinal symptoms were compared by the use of the non-parametric Wilcoxon signed rank test and adjusted using the Bonferroni-holm method. Significance was set to *p* < 0.05.

## 3. Results

### 3.1. Glucose, Insulin and C-Peptide

**Figure 1:**
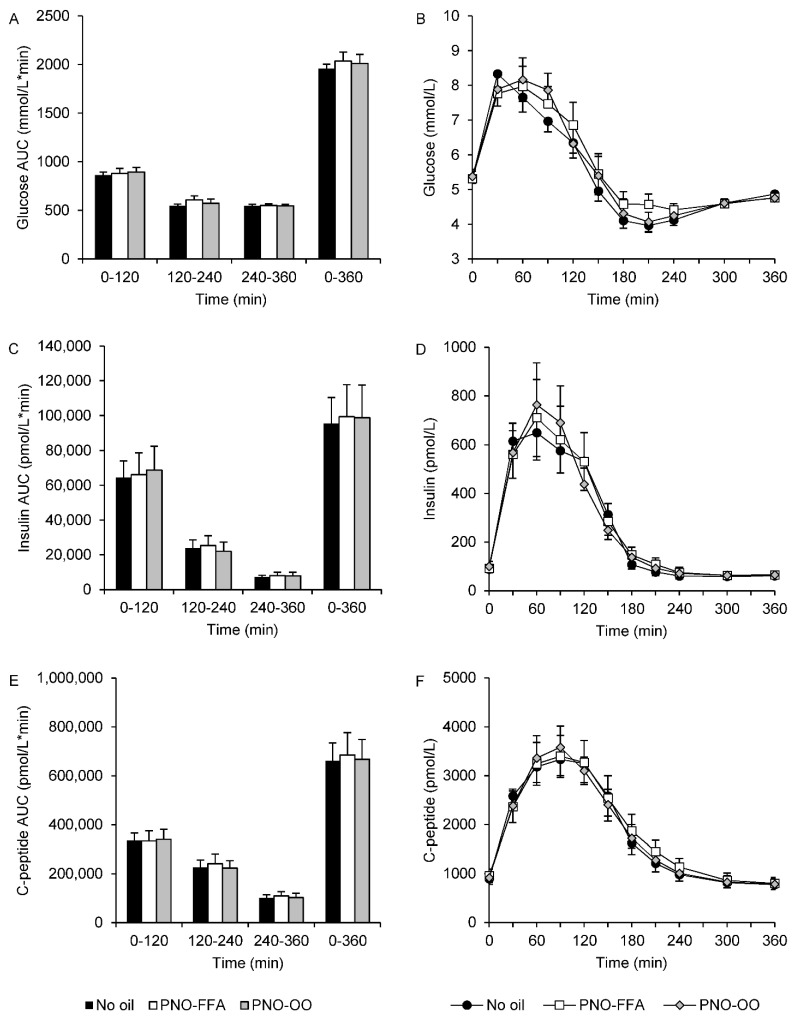
Glucose (**A**,**B**), insulin (**C**,**D**) and C-peptide (**E**,**F**) levels during a 6-h OGTT and their corresponding area under the curve calculated for 0–120, 120–240, 240–360 and 0–360 min in response to glucose alone (no oil, black bars and black circles), 6 g hydrolyzed pine nut oil (PNO-FFA, white bars and white squares) or 3 g hydrolyzed pine nut oil and 3 g olive oil (PNO-OO, gray bars and gray diamonds). Data are mean ± SEM.

There were no significant treatment differences in any of the calculated AUCs of glucose, insulin or C-peptide. Plasma glucose AUC_120–240_ tended to be slightly increased (~11%) after PNO-FFA versus no oil treatment (*p* = 0.098). OGTT response curves and corresponding AUCs are presented in Figure 1.

### 3.2. Hormone Secretion of GLP-1, GIP and Ghrelin

**Figure 2:**
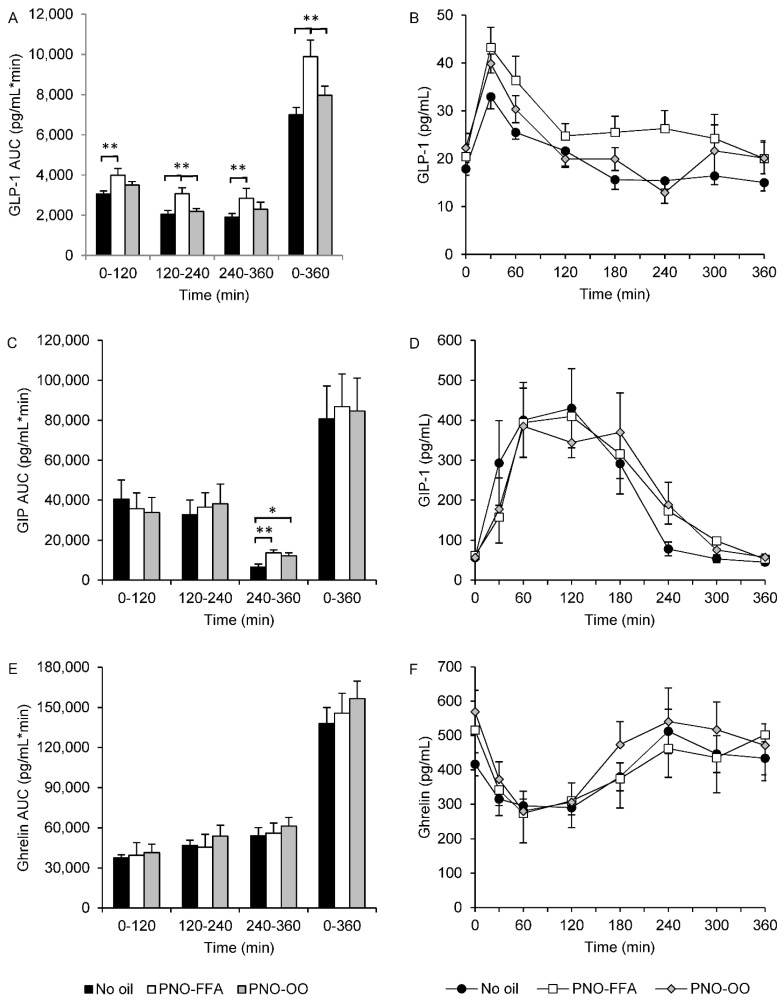
GLP-1 (**A**,**B**), GIP (**C**,**D**) and ghrelin (**E**,**F**) levels during a 6-h OGTT and their corresponding area under the curve calculated for 0–120, 120–240, 240–360 and 0–360 min in response to glucose alone (no oil, black bars and black circles), 6 g hydrolyzed pine nut oil (PNO-FFA, white bars and white squares) or 3 g hydrolyzed pine nut oil and 3 g olive oil (PNO-OO, gray bars and gray diamonds). Data are mean ± SEM. * *p* < 0.05, ** *p* < 0.01.

GLP-1 AUC_total_ and all two-hour AUC segments were increased (~30–50%) after PNO-FFA treatment compared to no oil (*p* < 0.01) (Figure 2A,B). Moreover, PNO-FFA also increased GLP-1 AUC_total_ and AUC_120–240_ (~6–14%), in comparison to PNO-OO (*p* < 0.01), whereas, GLP-1 AUC_0–120_ and AUC_240–360_ only tended to be increased (~14–21%) after PNO-FFA versus PNO-OO (*p* < 0.1) (Figure 2A,B). PNO-FFA and PNO-OO increased GIP AUC_240–360_ (~85–107%) compared to no oil (*p* < 0.01), and GIP AUC_total_ tended to be increased (~8%) after PNO-OO versus no oil (*p* = 0.066) (Figure 2C,D). None of the calculated ghrelin AUCs differed between treatments (Figure 2E,F).

### 3.3. FFA

PNO-FFA and PNO-OO reduced plasma FFA AUC_total_ compared to no oil (*p* < 0.01), and this was explained by reductions in the two last 2-h periods, AUC_120-240_ and AUC_240–360_ (all *p* < 0.01) (Supplementary Figure S2). There were no differences in plasma FFA between PNO-FFA and PNO-OO.

### 3.4. Insulin Sensitivity and Beta-Cell Function

There were no treatment differences in the calculated OGTT-derived indices of insulin sensitivity. Similarly, indices of beta-cell function showed no significant differences between treatments (Table 2).

### 3.5. Subjective Appetite

Satiety AUC_total_ increased after PNO-FFA and PNO-OO versus no oil (~25–28%; *p* ≤ 0.01 for all). None of the two-hour satiety segments differed between treatments. Hunger AUC_total,_ AUC_−30–120_ and AUC_240–360_ decreased after PNO-FFA versus no oil (~6–18%; *p* ≤ 0.031 for all). A decrease in hunger was also observed after PNO-OO versus no oil, though only for AUC_total_ and AUC_240–360_ (~6% for both; *p* < 0.05 for all). Fullness AUC_total,_ AUC_120–240_ and AUC_240–360_ increased after PNO-OO versus no oil (~24–47%; *p* ≤ 0.045 for all). An increase in fullness after PNO-FFA versus no oil was only observed for AUC_240–360_ (~46%; *p* = 0.048). Prospective food consumption was reduced after both PNO-FFA and PNO-OO for AUC_total_ and all two-hour segments (~6–18%; *p* ≤ 0.045 for all). Finally, overall well-being AUC_120–240_ was reduced after PNO-FFA versus no oil (~12%; *p* = 0.005). The two oil treatments did not differ in any of the assessed appetite symptoms. See Table 3, for all AUC appetite responses.

### 3.6. Gastrointestinal Tolerability

The delta value of flatulence decreased during the OGTT after no oil versus PNO-OO treatment (*p* = 0.023) and, also a tendency of a decrease after no oil versus PNO-FFA (*p* = 0.07) was observed. No treatment differences in delta values of bloating, diarrhea or constipation during the OGTT were observed and neither were there any treatment differences in gastrointestinal symptoms at 8PM or 8AM after the OGTTs. Outcomes on gastrointestinal tolerability are presented in Supplementary Table S1.

## 4. Discussion

Our major aim was to investigate whether combined intake of selected dietary lipids, could act in synergy to enhance GLP-1 secretion and consequently reduce appetite and improve insulin secretion and glucose tolerance in humans in vivo. This was done by testing the effects of delayed-release hydrolyzed pine nut oil, containing pinolenic acid, a potent dual FFA1/FFA4 agonist, in combination with olive oil, liberating the GPR119 agonist 2OG upon digestion, versus intake of hydrolyzed pine nut oil alone or no oil intake prior to an OGTT.

We detected no enhanced effect of combining hydrolyzed pine nut oil and olive oil on GLP-1 secretion when delivered to the small intestine by delayed release capsules. This is in contrast to a recent preclinical study showing a strong synergistic effect of activating both FFA1 and GPR119 in mouse colonic crypt cells on GLP-1 secretion [7]. One explanation may be the doses of pine nut oil and olive oil in our study, which may have been too small to activate FFA1 and GPR119 in vivo in humans. It has been found that 19 g of olive oil, which is converted to 7.7 g of 2OG upon complete digestion, increases postprandial GLP-1 secretion in humans versus no oil intake [18]. Our PNO-OO treatment containing 3 g of olive oil would contribute 1.2 g of 2OG upon digestion, only a 6th of the amount used by Mandøe et al. [18]. However, it has been demonstrated in humans that a relatively small dose of 2 g of 2OG given as a bolus through a nasojejunal tube was sufficient to increase GLP-1 levels from 0–25 min after administration [19]. This indicates that the administration of 2OG directly to the more distal part (jejunum) of the small intestine will be more efficient in activating GPR119 and hence increase GLP-1 secretion. In our study, pine nut oil and olive oil were delivered to the small intestine in delayed-release capsules, which has been shown to give a prolonged release profile in vitro [17]. Therefore, using this administration method the release site is much more uncertain as compared to both the preclinical study using a direct method by stimulating a specific cell line of colonic crypt cells [6] as well as the clinical study giving 2OG through a nasojejunal tube [19]. An early release would lead to hydrolysis in the upper intestinal tract and absorption before the receptor populations in the lower intestinal tract is reached, whereas a late release might lead to incomplete enzymatic hydrolysis and a lower amount of 2OG. It is therefore possible, that a less specific gastrointestinal release site together with a smaller dose diminished a potential stimulatory effect of 2OG in our study. Future studies addressing this issue by using a formulation that ensures release in the lower gut and possibly larger doses, preferably using purified 2OG to eliminate the dependence on enzymatic hydrolysis, are needed.

Importantly, we found a pronounced effect of 6 g PNO-FFA intake alone on GLP-1 AUC compared to both PNO-OO and no oil intake from 0–360 min of the OGTT. This effect was significant for all 2 h periods during the OGTT when compared to no oil, but only from 120–240 min compared to PNO-OO. These results are in line with our previous studies, which mainly demonstrated an increase in GLP-1 secretion 120–240 min after intake of PNO-FFA versus no oil [17]. Thus, the effect of delayed-release hydrolyzed pine nut oil on GLP-1 secretion seems to be a consistent and reproducible outcome. Interestingly, in the present study GLP-1 secretion was significantly increased already from 0–120 min of the OGTT. A similar pattern was seen in our previous study [17], however, these differences were less pronounced and did not reach significance. Ingestion of high doses of olive oil (30 mL) or oleic acid (0.88 g/kg body weight) have been reported to increase circulating GLP-1 levels suggesting a general effect of oil intake on GLP-1 secretion [30,31]. However, while intake of 6 g oil either as PNO-FFA or PNO-OO caused a similar reduction in plasma FFA from 120–360 min compared to no oil, only the double dose of hydrolyzed pine nut oil significantly increased GLP-1 levels. This support a specific effect of hydrolyzed pine nut oil on GLP-1 rather than a general effect of oils, at least at lower doses. Noteworthy, in this study we prolonged the acute OGTT test up to 360 min vs. 240 min in our previous study, as the previous results on GLP-1 indicated enhanced secretion after both 3 and 6 g PNO-FFA beyond the 240 min of testing. We were able to confirm this assumption, though the enhanced secretion slowly attenuates from 240–360 min.

Glucose tolerance was unaffected by the treatments in the present study. Thus, neither glucose, insulin, nor C-peptide responses showed any significant treatment differences. This is to some extent in contrast to our previous studies, in which we observed small reductions in glucose and insulin responses from 0–120 min of the OGTT after 3 and 6 g PNO-FFA intake versus no oil [17]. Overall, our results indicate less clear effects of PNO-FFA on glucose tolerance, and no enhancement when combining hydrolyzed pine nut oil with olive oil. Moreover, the present study shows that while treatment with PNO-FFA enhances GLP-1 secretion throughout the test, it does not convincingly increase insulin secretion or reduce glucose levels. The question is whether such effects can be expected in subjects with a normal glucose tolerance. Therefore, it would be important to test the acute effects of PNO-FFA in prediabetic or type 2 diabetic subjects in future studies, before potential beneficial effects on glucose and insulin secretion can be ruled out.

In this study, we observed no significant differences in ghrelin levels between any of the treatments. This is consistent with a previous study showing similar ghrelin responses after intake of 3 g doses of pine nut oil, pine nut oil free fatty acids or olive oil [28]. Thus, intake of these oils seems to affect ghrelin levels to a similar degree when given separately. However, in the present study, there were no differences between intakes of oils versus no oil, which is in contrast to what we have observed previously [17]. It is however consistent with the ghrelin receptor being primarily expressed in the upper gastrointestinal tract [32], whereas the delayed-release formulation targets the lower intestines. In comparison to our earlier studies, we experienced a substantial number of missing data on ghrelin levels as described above. This meant that the PNO-FFA treatment arm was reduced to seven subjects and the PNO-OO arm to six subjects, which may have reduced the power of the study and compromised the validity of the ghrelin results.

GIP secretion was unaffected by oil type from 0–240 min in this study. Across our own studies, we have not been able to reproduce consistent findings on GIP secretion. Previously we have reported that PNO-FFA 3 g versus no oil reduced GIP secretion from 0–120 min. and increased secretion from 120–240 min. But this result was not reproduced in our dose response study, and neither in this study. Moreover, we observed an increase in GIP secretion from 240–360 min after both oil types in this study, suggesting no distinct effect of oil type on GIP secretion. It could be speculated, that these inconsistent findings may be driven by the smaller abundancy of GIP secreting K-cells in the distal intestine (release site), combined with the less specific release profile of the capsules as described previously leading to different results. Therefore, further studies are necessary. Satiety and prospective food consumption were affected similarly with an appetite reducing effect after PNO-FFA and PNO-OO compared to no oil. Hunger was also reduced after both oil treatment versus no oil. PNO-FFA seemed to have the most pronounced effect with several two-hour segments being significant. This was opposite for fullness, where both oil treatments reduced fullness from 4–6 h versus no oil, while several AUCs were reduced after PNO-OO suggesting a more pronounced effect. Only PNO-FFA seemed to reduce overall well-being versus no oil from 2–4 h of the test. Despite a few differences, the overall picture was a similar appetite reducing effect of the two oil treatments. Despite an increase in the appetite suppressive hormone GLP-1 only after PNO-FFA, this did not significantly reduce the subjective appetite further in comparison to PNO-OO. It has been suggested that free fatty acids of pine nut oil, corresponding to our PNO-FFA treatment, is able to decrease appetite by reducing prospective food consumption in comparison to intake of olive oil alone [28]. However, we did not observe significant differences between the oil treatments, suggesting similar appetite reducing effects independent of oil type. Moreover, we did not include a pure olive oil arm in our study, and, therefore it is possible that the 3 g hydrolyzed pine nut oil in the PNO-OO treatment was sufficient to reduce appetite to the same extent as the 6 g PNO-FFA alone. This is consistent with the findings in our previous dose-response study, where we observed similar reductions in subjective appetite after intake of 3 and 6 g PNO-FFA, respectively [17]. Finally, an important limitation in our study is the lack of a blinded placebo treatment. The participants knew whether they received oil or not, which may have influenced the VAS results on appetite measures.

Gastrointestinal tolerability of the oil capsules was good. A reduction in flatulence during the OGTT after no oil compared to PNO-OO was observed. It is likely that this change reflects an increased level already prior to the OGTT, and therefore does not reflect an actual ameliorating effect of glucose intake compared to the oil treatments.

The major limitation of our study is the unblinded design in respect to no oil versus the two oil intake days. Moreover, the relatively small sample size made data susceptible for reduced validity when missing data occurred.

## 5. Conclusions

In conclusion, we did not observe an enhanced effect on GLP-1 secretion, glucose tolerance or appetite when combing oral intake of hydrolyzed pine nut oil (containing ~20% pinolenic acid) with olive oil (releasing 2OG) to potentially stimulate FFA1, FFA4 and GPR119 in the small intestine. Importantly, however, we could reproduce that hydrolyzed pine nut oil delivered to the small intestine by delayed-release capsules augments GLP-1 secretion. As GLP-1 is a known important gut hormone regulating glucose metabolism, subjective appetite and body weight it is relevant to further investigate whether treatment with hydrolyzed pine nut oil in delayed release capsules may be a future dietary supplement with potential beneficial effects on these outcomes in well-powered acute and long-term studies, and preferably in subject with prediabetes or overt type 2 diabetes.

## Figures and Tables

**Table 1:** Baseline characteristics (*n* = 9).

Age (Years)	54 ± 8
Sex, m/f (*n*)	4/5
Height (cm)	174 ± 9
Weight (kg)	105 ± 21
BMI (kg/m^2^)	34.4 ± 4.2
Fat mass (kg)	42 ± 10
Fat free mass (kg)	61 ± 17
Fat %	41 ± 8
Systolic blood pressure (mmHg)	135 ± 13
Diastolic blood pressure (mmHg)	85 ± 5
Plasma glucose (mmol/L)	5.3 ± 0.4
Creatinine (umol/L)	77 ± 10
Triglycerides (mmol/L)	1.2 ± 0.3
HDL-cholesterol (mmol/L)	1.5 ± 0.4
LDL-cholesterol (mmol/L)	3.1 ± 0.5
Total cholesterol (mmol/L)	5.1 ± 0.6
HOMA-IR *	3.0 ± 1.4

* Homeostatic model assessment of insulin resistance (HOMA-IR) calculated as: fasting glucose mmol/L × fasting insulin mU/L/22.5. Values are mean ± SD.

**Table 2:** OGTT based indices on insulin sensitivity and beta-cell function.

	No Oil	PNO-FFA	PNO-OO
**Insulin sensitivity**			
BIGTT_Si_	3.7 ± 0.9	4.0 ± 1.1	4.0 ± 0.8
MATSUDA	3.7 ± 0.8	4.0 ± 1.0	3.5 ± 0.7
**Beta-cell function**			
BIGTT_AIR_	4362 ± 641	4807 ± 1023	4847 ± 1109
CIR_30 min_	1791 ± 235	2026 ± 336	1760 ± 286

Values are mean ± SEM.

**Table 3:** Effects of PNO-FFA and PNO-OO on AUC appetite responses.

	No Oil	PNO-FFA	PNO-OO
**Satiety**			
AUC_−30–120 min_	5261 ± 303 ^1^	6183 ± 449	5917 ± 622
AUC_120–240 min_	2733 ± 359	3220 ± 285	3785 ± 511
AUC_240–360 min_	1527 ± 318	2297 ± 358	2260 ± 449
AUC_total_	9364 ± 789 ^1^	11,700 ± 822 *	11,962 ± 1390 *
**Hunger**			
AUC_−30–120 min_	9087 ± 137 ^1^	7448 ± 509 *	8586 ± 495
AUC_120–240 min_	8202 ± 529	8113 ± 442	8052 ± 429
AUC_240–360 min_	9690 ± 474	9123 ± 435 *	9157 ± 477 *
AUC_total_	27,380 ± 878 ^1^	24,684 ± 1027 *	25,794 ± 1123 *
**Fullness**			
AUC_−30–120 min_	4784 ± 654 ^1^	5487 ± 787	5343 ± 874
AUC_120–240 min_	2268 ± 363	2782 ± 459	3245 ± 687 *
AUC_240–360 min_	1317 ± 279	1920 ± 376 *	1930 ± 521 *
AUC_total_	8499 ± 1380 ^1^	10,188 ± 1451	10,518 ± 1965 *
**Prospective food consumption**			
AUC_−30–120 min_	9815 ± 560 ^1^	8082 ± 759 *	8819 ± 753 *
AUC_120–240 min_	9098 ± 436	8348 ± 501 *	8387 ± 603 *
AUC_240–360 min_	9797 ± 496	8993 ± 630 *	9227 ± 687 *
AUC_total_	28,850 ± 1442 ^1^	25,423 ± 1658 *	26,433 ± 1847 *
**Overall well-being**			
AUC_−30–120 min_	12,111 ± 877 ^1^	10,256 ± 1187	11,184 ± 1073
AUC_120–240 min_	9150 ± 676	8095 ± 953 *	9022 ± 769
AUC_240–360 min_	8486 ± 793	8070 ± 918	9000 ± 713
AUC_total_	30,004 ± 1993 ^1^	26,421 ± 2981	29,206 ± 2496

VAS appetite responses calculated as area under the curve (AUC, mm*min). Values are mean ± SEM. Superscript numerals indicate number of values missing completely at random. * *p* < 0.05 versus no oil.

## Data Availability

The data presented in this study can be requested from the corresponding author.

## Supplementary Materials

The following are available online at https://www.mdpi.com/article/10.3390/nu13103407/s1, Figure S1: Study design, Figure S2: Free fatty acids (FFA) levels during a 6-h OGTT, Table S1: Changes in gastrointestinal tolerability during the OGTT, at 8PM after the OGTT and at 8AM after the OGTT.
