# Supplementary material for: Effects of Delayed-Release Olive Oil and Hydrolyzed Pine Nut Oil on Glucose Tolerance, Incretin Secretion and Appetite in Humans
Source: Nutrients. 2021 Sep 27;13(10):3407. doi: 10.3390/nu13103407 (PMC8538272; doi:10.3390/nu13103407)
Supplement: Supplementary file 1 [file nutrients-13-03407-s001.zip › nutrients-1372577-Supplementary.pdf]

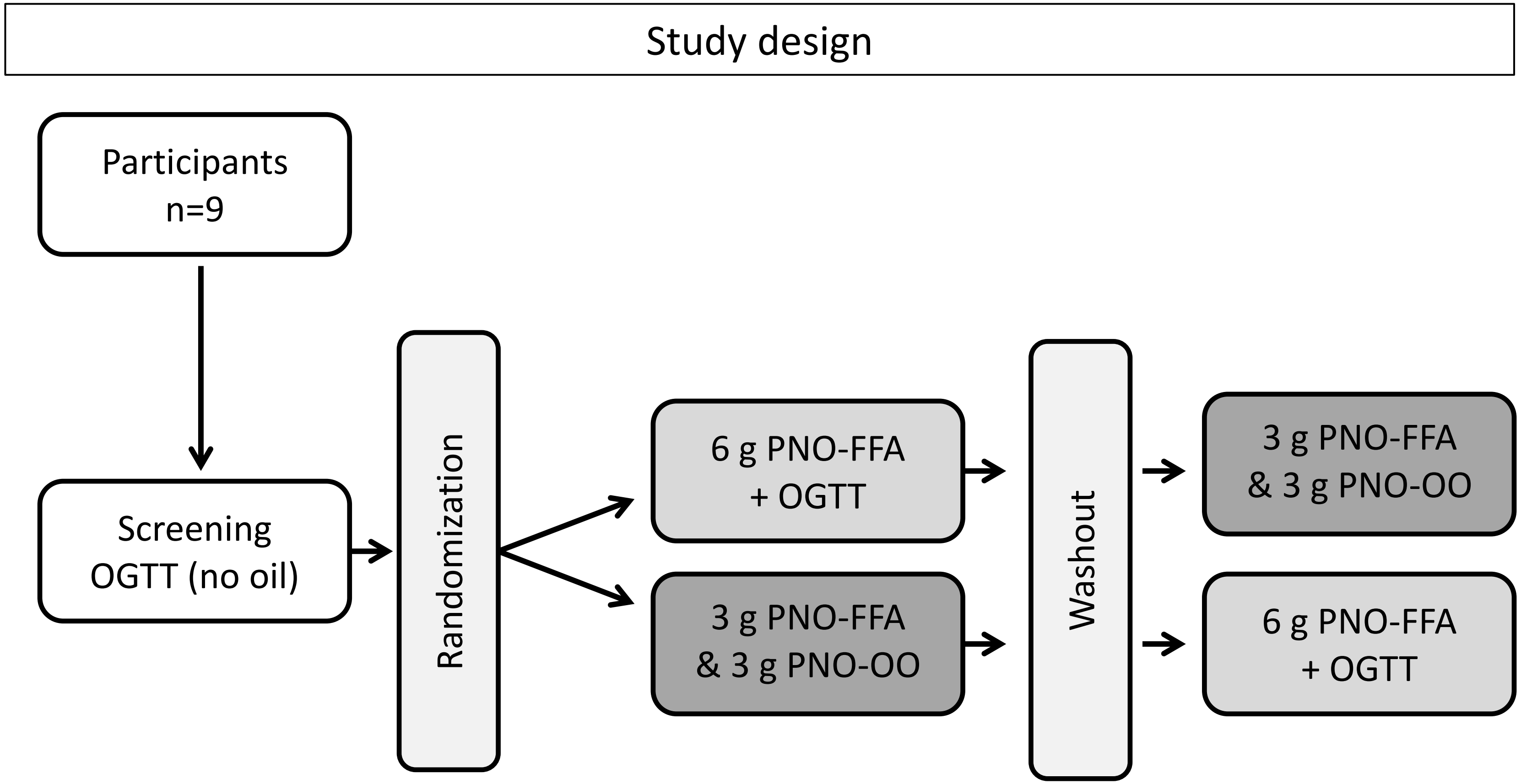

Figure S1. Study design

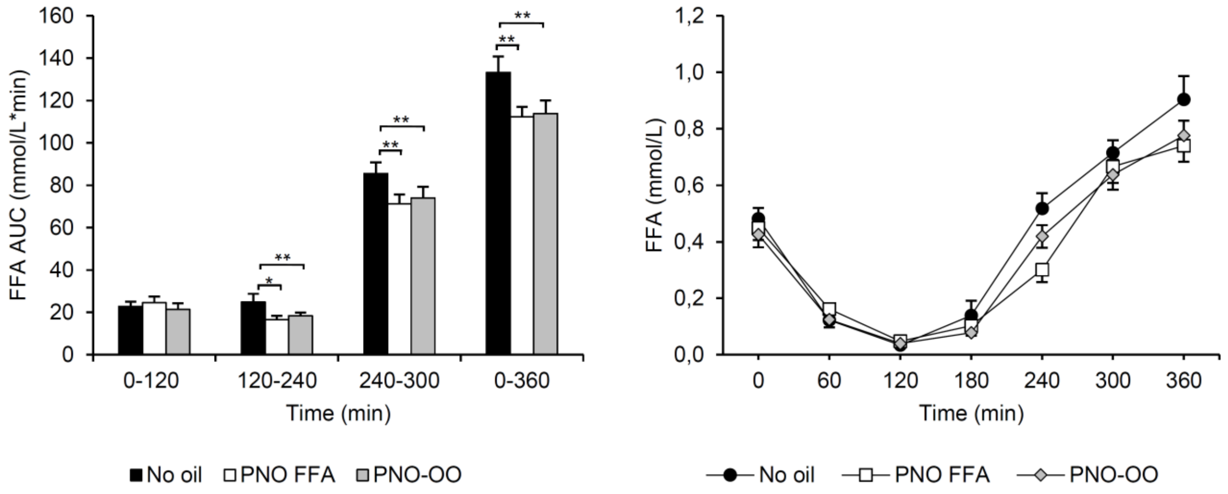

Figure S2. Free fatty acis (FFA) levels during a 6-hours OGTT and their corresponding area under the curve calculated for 0-120, 120-240, 240-360 and 0-360 min in response to glucose alone (no oil, black bars and black circles), 6 g hydrolyzed pine nut oil (PNO-FFA, white bars and white squares) or 3 g hydrolyzed pine nut oil and 3 g olive oil (PNO-OO, gray bars and gray diamonds). Data are mean ± SEM. *$p<0.05$, **$p<0.01$

Table S1. Delta values of gastrointestinal tolerability during the OGTT, and mean values at 8PM after the OGTT and at 8AM after the OGTT.

	No oil	PNO-FFA	PNO-OO
**Flatulence**			
Δ 0-360 min	-15.4 ± 6.3	0.1 ± 4.6	2.1 ± 2.4*
8 PM evening after OGTT	11.2 ± 3.2	11.4 ± 3.0[1]	12.2 ± 2.5
8 AM morning after OGTT	11.9 ± 3.3	12.3 ± 2.3[1]	10.2 ± 3.50
**Bloating**			
Δ 0-360 min	0.2 ± 1.2	0.3 ± 1.1	1.4 ± 2.70
8 PM evening after OGTT	11.2 ± 3.2	2.6 ± 1.3[1]	3.0 ± 1.2
8 AM morning after OGTT	5.7 ± 2.5	1.1 ± 0.4[1]	2.8 ± 1.5
**Nausea/vomiting**			
Δ 0-360 min	5.0 ± 3.8	-1.7 ± 5.9	-1.3 ± 1.2
8 PM evening after OGTT	4.6 ± 2.5	5.8 ± 2.9[1]	4.4 ± 3.3
8 AM morning after OGTT	3.2 ± 2.0	1.0 ± 0.3[1]	1.4 ± 0.4
**Diarrhea**			
Δ 0-360 min	0 ± 0.4	10.3 ± 8.7	0.1 ± 0.2
8 PM evening after OGTT	7.2 ± 6.6	14.3 ± 7.1[1]	1.3 ± 0.5
8 AM morning after OGTT	0.8 ± 0.4	2.3 ± 1.3[1]	1.0 ± 0.3
**Constipation**			
Δ 0-360 min	-2.7 ± 2.0	0.7 ± 0.7	0 ± 0.50
8 PM evening after OGTT	0.8 ± 0.3	6.0 ± 4.8[1]	2.6 ± 1.5
8 AM morning after OGTT	10.9 ± 10.1	4.6 ± 3.1[1]	3.8 ± 2.4
**Abdominal pain**			
Δ 0-360 min	2.0 ± 2.0	0.6 ± 2.1	-0.1 ± 0.8
8 PM evening after OGTT	1.1 ± 0.5	3.8 ± 2.5[1]	1.6 ± 0.5
8 AM morning after OGTT	6.2 ± 5.2	0.9 ± 0.5[1]	1.2 ± 0.6

Gastrointestinal symptoms measured by use of visual analog scales (VASs, mm). Values are mean ± SEM.

Superscript numerals indicate number of missing values. *p<0.05 versus no oil.
